# Feasibility of Holographic Team Training Simulation: An Information Technology (IT) Perspective for Healthcare and Educational Institutions

**DOI:** 10.7759/cureus.65380

**Published:** 2024-07-25

**Authors:** Maria Bajwa, Melissa Morris, Wajeeha Ghias, Adam Linzels

**Affiliations:** 1 Health Professions Education, School of Healthcare Leadership, MGH Institute of Health Professions, Boston, USA; 2 Health Care Sciences, Dr. Pallavi Patel College of Health Care Sciences, Nova Southeastern University, Fort Lauderdale, USA; 3 Leadership and Management Studies, National Defence University, Islamabad, PAK; 4 Audio Visual Architecture, Engineering and Integration, Nova Southeastern University, Fort Lauderdale, USA

**Keywords:** information technology (it), hologram, holography, unified theory of acceptance and use of technology (utaut), teamstepps training, feasibility study, holographic display technology (hdt)

## Abstract

Introduction

This study examines the feasibility and practicality of holographic display technology (HDT) in health professions education from an information technology (IT) support staff perspective. Considering a lack of feasibility studies for introducing newer technologies, it focuses on feasibility’s acceptance and practicality dimensions during a simulation-based team training workshop.

Method

A multimethod design feasibility study assessed the acceptability and practicality of HDT for the IT staff through a Unified Theory of Acceptance and Use of Technology (UTAUT)-based survey and a focus group discussion after a team training simulation workshop.

Results

Quantitative results showed a reliability coefficient (α=0.83) and a positive correlation between facilitating conditions (FC) and effort expectancy (EE), self-efficacy (SE) and social influence (SI), SI and attitude toward technology (AT), SE and attitude to use, and behavioral intention (BI) and EE. Negative correlations included SE and performance expectancy (PE), comfort with technology and FC, comfort and anxiety, and attitude to use and experience. Qualitative findings yielded four key themes from the focus group discussions that corroborated the quantitative findings.

Discussion

The study findings highlight the promising potential for HDT feasibility in educational settings. Future research should extend to diverse contexts to validate these preliminary findings and explore broader educational applications.

## Introduction

With the advent of virtual reality and e-learning, educational institutions and healthcare facilities have been exploring more immersive and interactive mediums for teaching, learning, and training. Among current innovations, holographic display technology (HDT) is a potentially transformative tool; it offers 3D interactive visuals that promise to deepen comprehension and engagement among learners [1,2]. Several studies have explored the adoption of holographic technologies in education, providing insights into the potential challenges and benefits of implementing such technologies [1-4].

Despite its potential, the broader adoption of this technology in educational and healthcare settings faces significant feasibility challenges at organizational levels, such as a lack of technology infrastructure and faculty acceptance [2]. However, our literature search did not produce any studies that addressed the feasibility of HDT. There is a pressing need to explore and evaluate the feasibility of such advanced technology, especially regarding its acceptability and practicality [4]. Our study focused on bridging the gap in understanding HDT’s practical application and integration into healthcare education from the perspective of information technology (IT) personnel. Therefore, this study aimed to assess HDT’s acceptability and practicality constructs of the feasibility in IT at a Southern Florida university facilitating a teamwork training simulation across multiple locations. 

Overview of holographic technology

The HDT has been in the medical field for quite some time [4]. It has recently garnered attention for its potential in educational environments due to its immersive and interactive capabilities [1]. It enables interaction with remote holographic subjects and 3D anatomical models, practicing complex medical procedures, and analyzing virtual patient cases. Holographic displays provide a means to present and view pre-recorded content or live video feeds as a three-dimensional image, creating the effect of visual depth while offering immersive experiences beyond traditional technology [1].

The hologram unit's 3D effect employs three visual planes within a rectangular display box, featuring an 86” diagonal clear front vertical display as the first plane; the second plane is the clear space to the back wall, creating true visual depth between the primary display and the third plane, which is the vertical back wall of the physical display case. Internal light panels on the enclosure's sides, top, and back illuminate planes two and three, creating a lightbox effect that evenly distributes light, defining the depth of the enclosure and enhancing the 3D effect. The visual volume, tricking the eye into perceiving depth, is achieved through video production techniques, capturing pre-recorded and live footage using a camera, white background, and offset light for shadow casting, creating a lightbox effect [1,5].

The newer HDT's videoconferencing capability allows real-time virtual presence and two-way communication. Touch displays enable interaction with holographic content, which is especially useful for volumetric images and manipulation of 3D objects for better understanding. Displays vary in size, enhancing realism based on the visual volume and creating life-size objects to convince viewers. The technology's capacity to simulate real-life scenarios in a controlled environment is particularly beneficial for training healthcare professionals, where hands-on experience is crucial [2].

Feasibility study

A feasibility study determines whether a more extensive trial or intervention will be successful and should be considered for that particular intervention [6]. This research evaluated the feasibility of the HDT for IT personnel, focusing specifically on acceptability and practicality pertinent to the context as the first steps in the institutional adoption of any technology [6,7]. Other aspects, efficacy, demand, implementation, adaptation, integration, and expansion, were deemed less relevant for the immediate scope of this paper as these aspects are evaluated in the later stages of technology adoption [7]. Acceptability was assessed using a Unified Theory of Acceptance and Use of Technology (UTAUT)-based survey to understand the IT’s response [8]. The survey examined performance expectancy (PE), effort expectancy (EE), social influence (SI), and facilitating conditions (FC) on behavioral intentions (BI) and attitude toward technology (AT) (see Table 5 and Table 6 of Appendices for UTAUT definitions and survey details) [8]. Practicality was addressed through focus group discussion with IT personnel, evaluating the intervention's ease of use and its demand on institutional support [7]. The study gauged the intervention's potential success within organizational constraints and the specific technological setup by focusing on these areas (see Table 5 and Table 6 of Appendices). Data were collected from learners, faculty, and IT support staff during the workshop. However, this article focuses on IT personnel's unique and underreported insights, which are crucial for educational interventions. Results from other groups will be addressed in subsequent studies.

TeamSTEPPS

We assessed the feasibility of the HDT for the IT team who managed the technology during the Team Strategies and Tools to Enhance Performance and Patient Safety (TeamSTEPPS™) workshop for healthcare learners. TeamSTEPPS is a foundational tool designed to improve patient outcomes by enhancing teamwork and communication in healthcare settings [9]. We chose to conduct this feasibility study with TeamSTEPPS because of 1) its foundational value in healthcare, 2) the university offers it routinely to its affiliates, which allowed us to conduct this training in a non-traditional way using HDT without additional costs to the university, and 3) the IT conducted this training historically using other technologies, leading to their familiarity with the routine, procedure, and apparatus used to facilitate this simulation. 

Ethical considerations

The ethical review board of Nova Southeastern University deemed this study exempt (NSUIRB #2023-223). The study participants signed the consent form and were made verbally aware of their rights at the beginning of the intervention.

## Materials and methods

Study design

**Figure 1:**
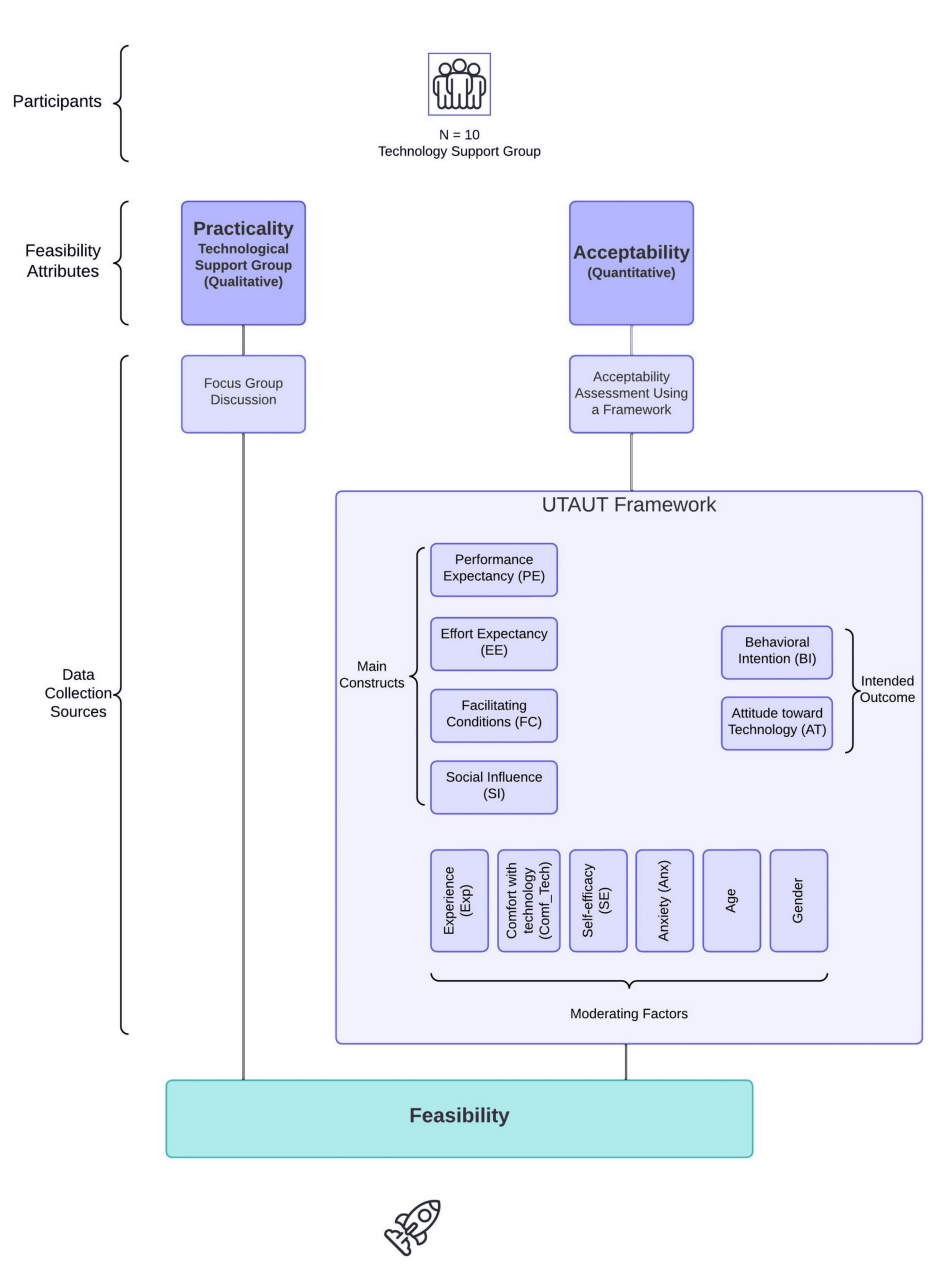
The flow chart of feasibility research for the IT group Flow chart outlining the feasibility research process for the IT group. IT, information technology

This study used a multimethod design to explore the feasibility of holographic technology for the IT team during TeamSTEPPS training [10]. The multimethod approach helps explore complex real-life applications involving multiple intersecting factors, gathering qualitative and quantitative data [10,11] (Figure 1).

Participants

The study participants comprised university IT personnel assigned to support the activity. Their participation in the facilitation was mandatory as they were university employees, and facilitation was part of their jobs; however, their participation in the research was voluntary. The inclusion criteria were to be part of the university's technological support system and involvement in preparing and delivering this simulation. 

Process

The structured TeamSTEPPS training workshop lasted two days and featured two three-hour sessions of didactic presentations, discussions, simulations, and evaluations. The simulations followed evidence-based practices, specifically the distance simulation guidelines for all simulation phases, including prebriefing, simulated participant (SP) activities, and debriefing [12-14].

The design was more time-intensive than previous workshops due to the additional steps required, necessitating close collaboration between simulation educators and the IT team. Considering the extra time required, this process was extended by approximately three business days. This included multiple iterations to design the scenario while maintaining the integrity of the goals within the technology's constraints, conducting multiple dry runs to ensure the technology functioned correctly, and troubleshooting any issues. Additionally, the logistics of setting up and moving equipment into and out of the space further added to the overall time commitment.

**Figure 2:**
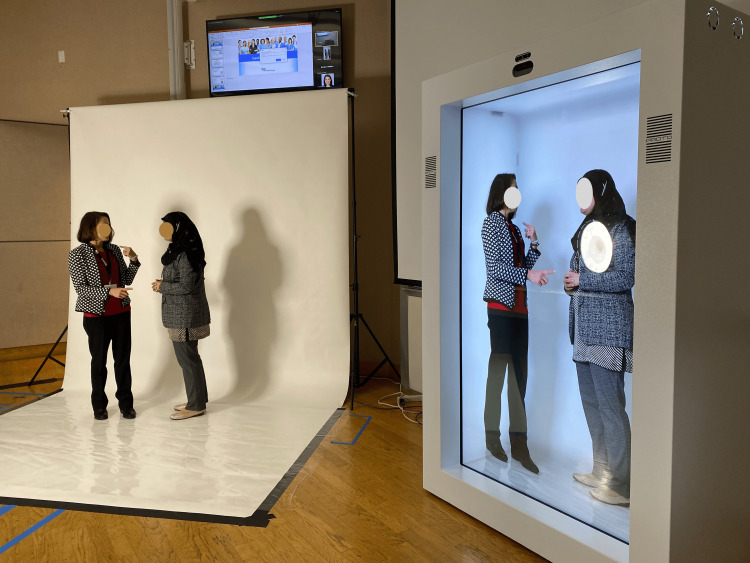
Lightbox effect during holoprojection adding depth to the SPs in the HDT box SPs, simulated participants; HDT, holographic technology display

**Figure 3:**
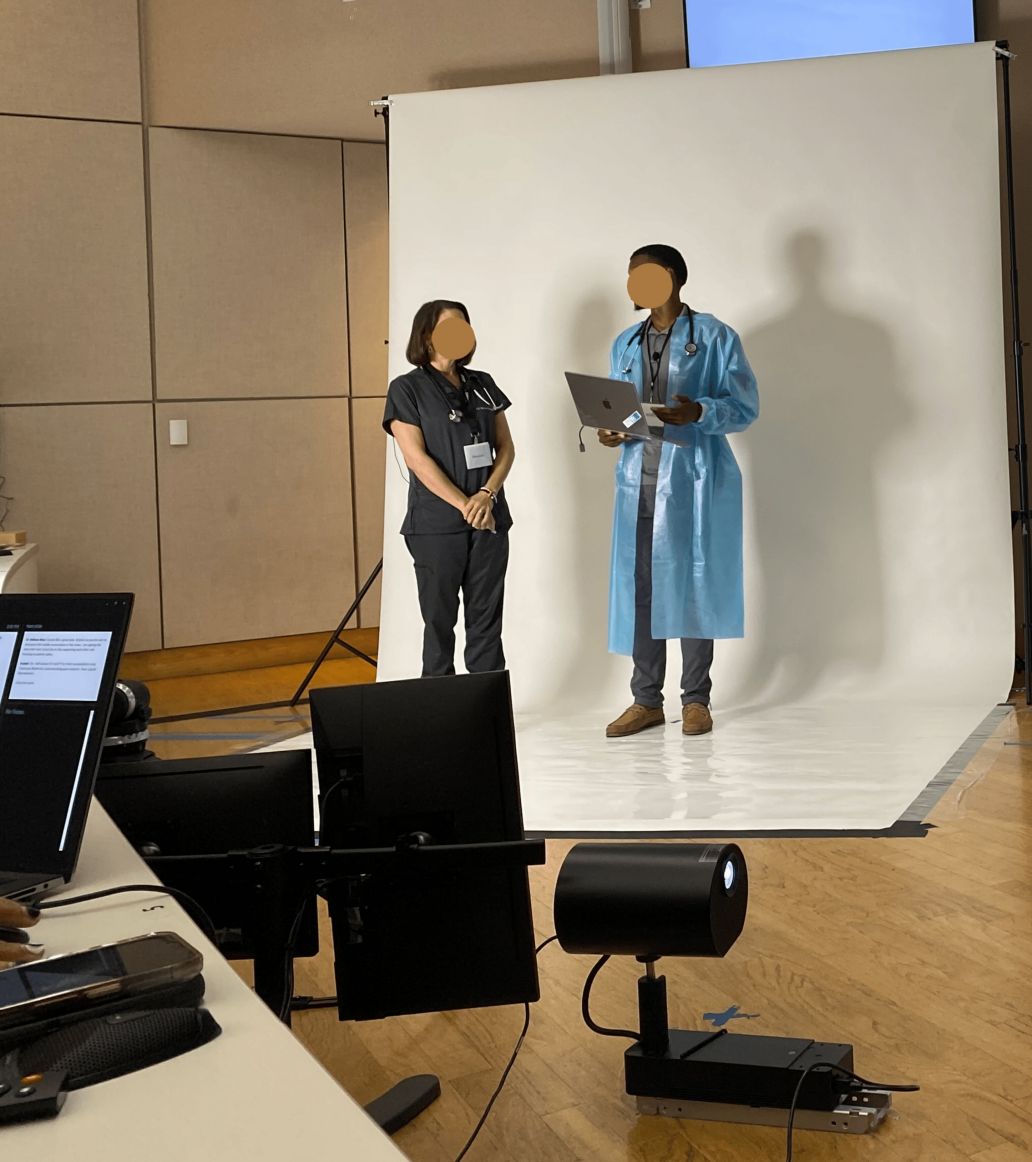
Holoprojection/live projection of SPs portraying a physician and a nurse in a TeamSTEPPS simulation (a sender unit view) SPs, simulated participants

**Figure 4:**
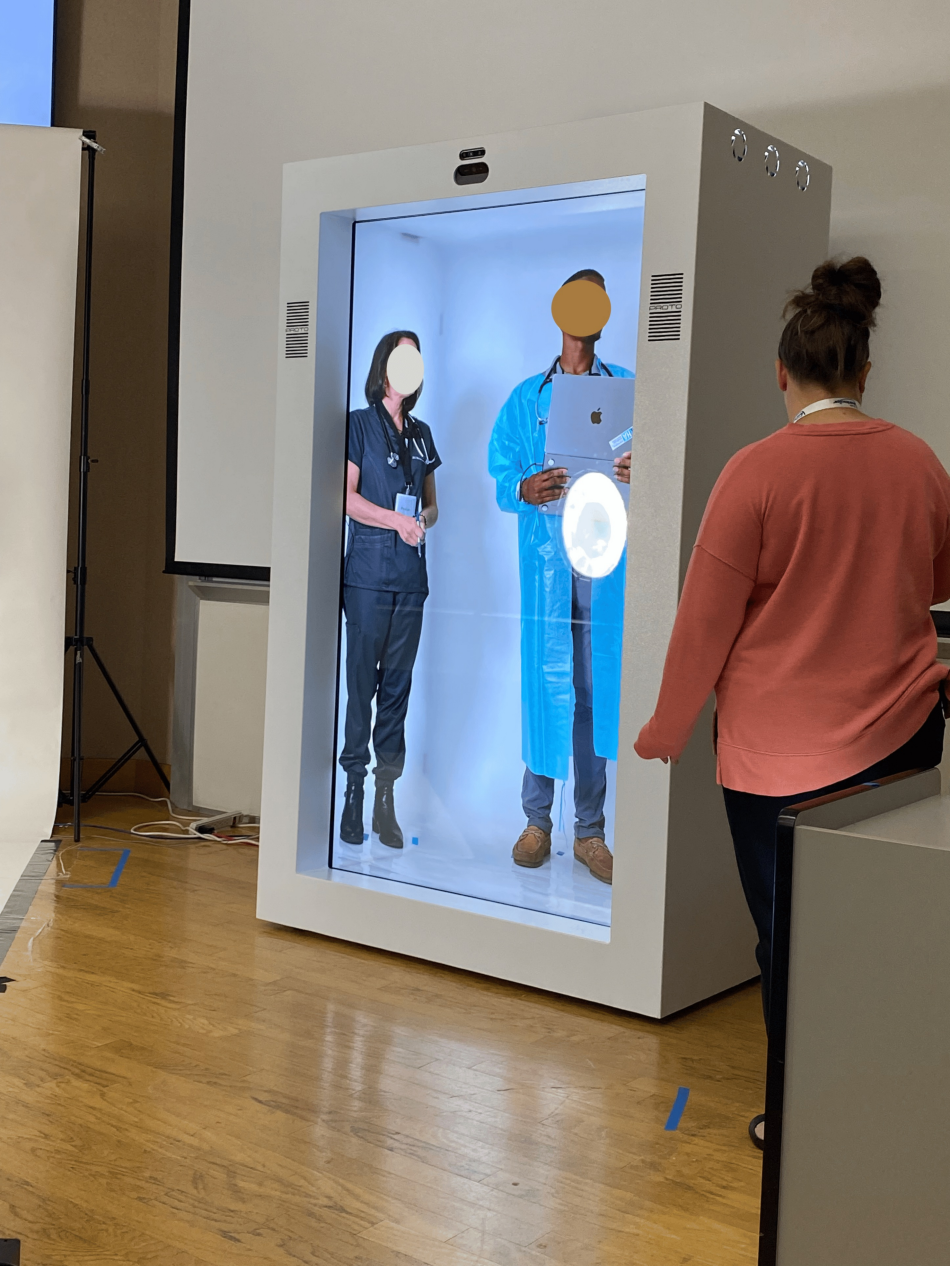
Holoprojection/live projection of SPs portraying a nurse and a social worker in TeamSTEPPS simulation (a receiver unit view) SPs, simulated participants

On the first workshop day, we used a pre-recorded scenario, while on the second day, the scenario was live-streamed using SPs. At the end of the second day, IT team members completed the UTAUT survey and participated in a focus group discussion to evaluate the session’s effectiveness and gather feedback (Figures 2-4).

The HDT setup

Since 2016, this university has employed holographic displays for virtual presence and anatomy lessons, starting with 55" screens and progressing to 86" models for a more life-like experience. This study used two proto epic holographic displays in three ways: 1) for live remote virtual holographic presence between both campuses, 2) for displaying pre-recorded and rendered holographic content at each campus, and 3) for an interactive scenario of interprofessional simulation where SPs in an offsite virtual holographic environment communicated live with the onsite SPs [15] (Figure 3).

In addition, some remote off-campus participants joined via Zoom videoconferencing and viewed the holographic scenarios through an audience view [16]. The HDT, integrated with local audio-visual systems and Zoom, allowed live two-way communication and engagement. This footage was live-streamed via Zoom to the holographic displays at each campus. Audio-visual IT staff facilitated this setup, tailoring it to the specific needs of TeamSTEPPS training programs.

Data collection

Post-session, participants completed a UTAUT survey, rating their attitudes and intentions toward holographic technology on a one to five Likert scale. The UTAUT survey, as adapted from Venkatesh et al., has robust psychometric properties [8]. This validation ensures the survey's reliability and validity, confirming that it accurately measures user acceptance and technology usage, consistent with the underlying theoretical constructs (see Table 5 of Appendices). It uses four key constructs to determine user acceptance and usage behavior with several modifying factors (Figure 1). 

Qualitative insights were drawn from a focus group discussion, exploring user experiences and feedback (Table 1), whose questions were guided by UTAUT and debriefing frameworks. Data was stored securely on the university's cloud and accessed only by MB and MM, with all identifying information removed to ensure participant anonymity.

**Table 1:** Questions asked in the focus group discussion to the IT personnel

Questions asked in the focus group discussion to the IT personnel
How are you feeling about the execution of the hologram activity?
We have been preparing for this activity for some time. What are your overall thoughts about that?
How much of your workload increased in planning, preparing, and conducting this activity using holographic technology?
Did you have to postpone your regular work duties, jobs, and tasks to deliver this activity using holographic technology?
What benefits do you see from this technology for this institute?
What benefits do you see from adapting this technology for your department?
Can this technology be an asset or a barrier in learning/teaching?
What challenges did you face during the preparation of this session? How should we have mitigated it?
Is this technology worth using in terms of institutional resources?

Quantitative data analysis

**Figure 5:**
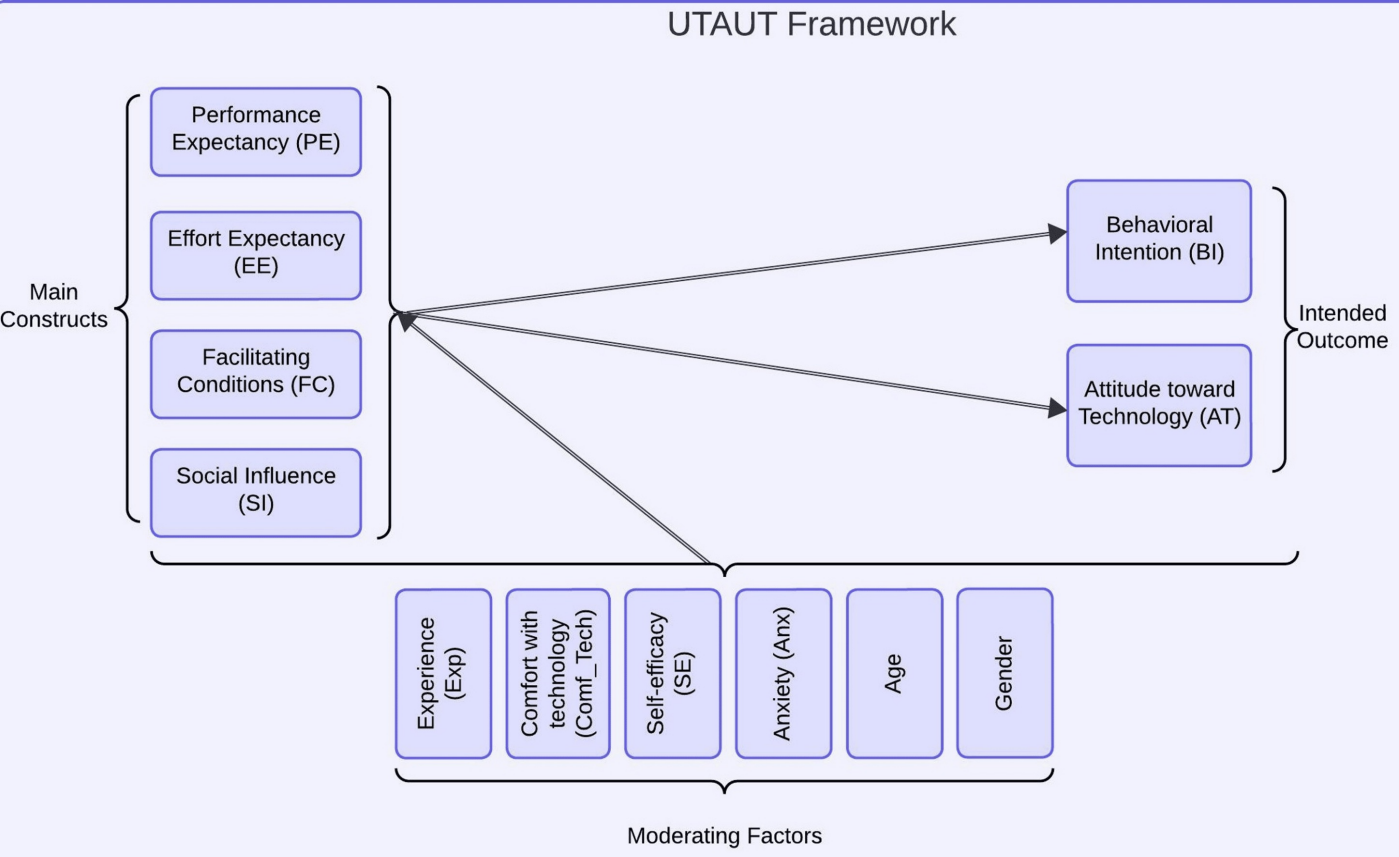
Model tested for a feasibility study for IT personnel IT, information technology; UTAUT, Unified Theory of Acceptance and Use of Technology

Quantitative software was used to perform the quantitative analysis [17,18]. We conducted descriptive statistics to investigate normal distribution and differences in age and gender. Cronbach's alpha was used to assess internal consistency. Pearson correlation analysis assessed relationships and their strength and direction, if present, between the dependent variables (DV) and independent variables (IV). The DV included BI and AT, while the IV included PE, EE, SI, and FC. Linear regression analysis was performed to examine the contribution of self-efficacy (SE), experience in years (Exp), and comfort with technology to the correlations identified through Pearson's correlation analysis (Figure 5). 

Qualitative data analysis

Following Braun et al. methodology, MB and MM performed a thematic analysis of the focus group transcript [19]. This approach was selected based on its prevalence in UTAUT studies, as identified in a systematic review [20]. They familiarized themselves with the data through repeated reviews and identified initial codes, which they organized into themes. During several discussions, they resolved discrepancies and reached a consensus on the themes' definitions and relevance to the study's goals, ensuring quality and reflexivity in the analysis through a continued process of self-examination and critical reflection on their contributions to the research process and how these influence every stage of analysis [21].

## Results

Demographics

Ten IT department participants, 80% male and 20% female, aged 26 to 47, with one to 18 years of professional experience, participated in this research. Roles included audiovisual systems, IT support, programming, and management. Participants reported comfort with technology at 3.7 out of five, troubleshooting ability at 3.8, and willingness to use new technology at 3.1.

Quantitative results 

Descriptive statistics revealed a non-normal distribution of the data. The Cronbach's alpha coefficient for internal consistency among the variables was reported to be 0.83 (lower bound 0.70 and upper bound 0.96), indicating good reliability [22].

**Figure 6:**
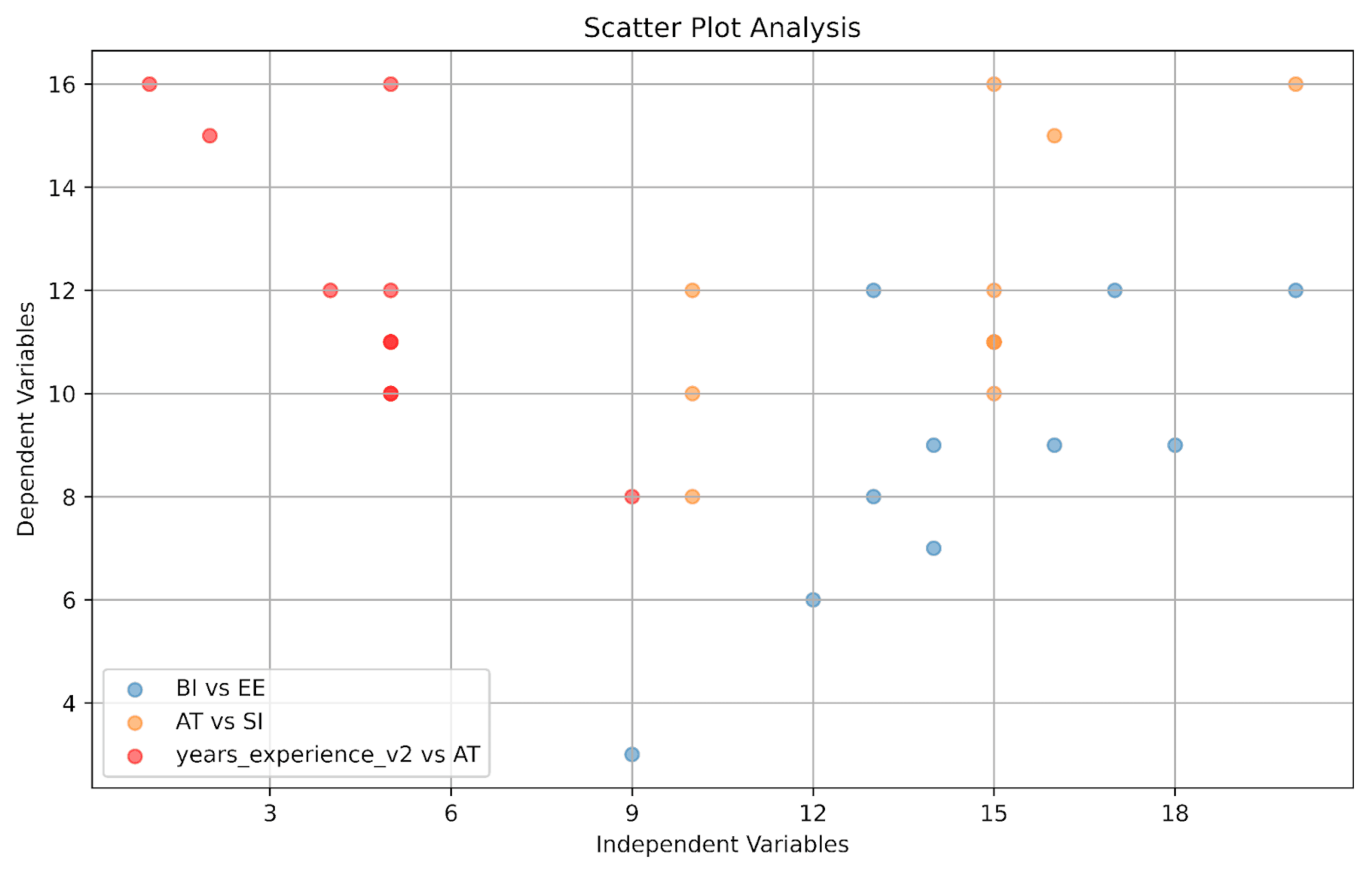
Scatter plots of selected variables This plot visualizes the relationships: BI and EE are shown in blue. Attitude and SI are orange. Attitude and years of experience (years_experience_v2) are shown in red. BI, behavioral intention; EE, effort expectancy; SI, social influence

These scatter plots (Figure 6) visually represent the correlations identified earlier, such as the positive correlation between BI and EE, as well as AT and SI. The negative correlation between years of experience and AT is highlighted in red, showing that more experience might be associated with less favorable attitudes toward the technology. 

Pearson’s Correlation

The Pearson correlation analysis revealed relationships among the variables. PE correlates negatively with EE, SI, FC, SE, anxiety, and experience, yet it correlates positively with comfort with technology. EE positively correlates with FC, BI, SI, and AT, suggesting that higher EE enhances BI and attitudes toward technology (Table 2).

**Table 2:** Pearson’s correlations among study variables (N=10) Values in the right upper section of the table are the mirror images of the left lower section diagonally - a common phenomenon in SPSS outputs; therefore, they are omitted and represented by dashes (-) *Correlation is significant at the 0.05 level (2-tailed). **Correlation is significant at the 0.01 level (2-tailed). PE, performance expectancy; EE, effort expectancy; SI, social influence; FC, facilitating conditions, AT, attitude towards technology, BI, behavior intentions, SE, self-efficacy, Anx, anxiety, Tech_Comf, comfort with technology, Exp, experience

Variables		PE	EE	SI	FC	SE	AX	TEC	Exp	Gender	Age	BI	AT
PE	Pearson Correlation	1	-	-	-	-	-	-	-	-	-	-	-
	Sig. (2-tailed)	-	-	-	-	-	-	-	-	-	-	-	-
EE	Pearson Correlation	-.539	1	-	-	-	-	-	-	-	-	-	-
	Sig. (2-tailed)	.108	-	-	-	-	-	-	-	-	-	-	-
SI	Pearson Correlation	-.331	.414	1	-	-	-	-	-	-	-	-	-
	Sig. (2-tailed)	.351	.234	-	-	-	-	-	-	-	-	-	-
FC	Pearson Correlation	-.505	.662^*^	.601	1	-	-	-	-	-	-	-	-
	Sig. (2-tailed)	.137	.037	.066	-	-	-	-	-	-	-	-	-
SE	Pearson Correlation	-.677^*^	.567	.657^*^	.399	1	-	-	-	-	-	-	-
	Sig. (2-tailed)	.032	.087	.039	.254	-	-	-	-	-	-	-	-
Anx	Pearson Correlation	-.427	.167	.490	.547	.377	1	-	-	-	-	-	-
	Sig. (2-tailed)	.219	.645	.151	.102	.283	-	-	-	-	-	-	-
Comf_Tech	Pearson Correlation	.740^*^	-.382	-.450	-.741^*^	-.586	-.710^*^	1	-	-	-	-	-
	Sig. (2-tailed)	.014	.276	.191	.014	.075	.021	-	-	-	-	-	-
Exp	Pearson Correlation	-.415	-.007	-.042	-.044	.123	.386	-.328	1	-	-	-	-
	Sig. (2-tailed)	.267	.986	.915	.910	.753	.304	.389	-	-	-	-	-
Gender	Pearson Correlation	-.210	.345	-.230	.382 u	-.299	-.051	-.084	.225	1	-	-	-
	Sig. (2-tailed)	.560	.328	.523	.276	.402	.889	.817	.560	-	-	-	-
Age	Pearson Correlation	-.264	-.232	-.025	-.241	.087	.357	-.172	.876^**^	.141	1	-	-
	Sig. (2-tailed)	.461	.519	.944	.503	.810	.312	.634	.002	.697	-	-	-
BI	Pearson Correlation	-.185	.749^*^	.586	.531	.500	.018	-.156	-.012	.308	-.095	1	-
	Sig. (2-tailed)	.608	.013	.075	.115	.141	.960	.668	.975	.386	.795	-	-
AT	Pearson Correlation	-.234	.616	.684^*^	.569	.658^*^	.098	-.303	-.681^*^	-.271	-.619	.565	1
	Sig. (2-tailed)	.515	.058	.029	.086	.038	.787	.395	.043	.449	.056	.089	-

SI positively affects FC and SE, indicating that increased SI can improve FC and SE. FC are positively associated with BI and AT, reinforcing the role of supportive environments in shaping technology usage intentions and attitudes. SE negatively correlates with PE but positively correlates with SI and AT, suggesting that higher SE contributes to positive attitudes toward technology despite lower PE.

Conversely, anxiety and comfort with technology negatively correlate with FC and AT, respectively, highlighting that anxiety and discomfort with technology can hinder the adoption of new technologies. Experience shows a positive correlation with age, emphasizing that more experienced individuals are typically older. This trend is noteworthy as gender does not significantly correlate with other variables. Moreover, BI is positively linked with both EE and AT. At the same time, AT correlates positively with SI and SE and negatively with experience. These correlations highlight the complex dynamics between experience, attitudes, and technology adoption, supported by the p-values provided in the analysis. 

*Linear Regression Analysis of Variables*****

The linear regression analyses were computed in SPSS 25 to test the linear relationship among variables (Table 3). The EE has a positive relationship with BI (β=0.82, p≥ 0.04), meaning a percentage increase in EE will increase BI. The rest of the study variables do not contribute to any change in BI. No variables significantly impacted AT, as all p-values exceeded 0.05.

**Table 3:** Results of linear regression for BI DV, dependent variables; BI, behavior intention

DV BI linear regression, N=10								
Variables		Unstandardized coefficients		Standardized coefficients	t	Sig.	95.0% confidence interval for B	
		B	Std. error	Beta			Lower bound	Upper bound
1	Constant	-2.769	1.999		-1.385	.225	-7.909	2.371
	PE	.397	.309	.353	1.287	.254	-.396	1.191
	EE	1.246	.477	.824	2.613	.047	.020	2.472
	FC	-.175	.717	-.085	-.244	.817	-2.017	1.668
	SI	.622	.424	.412	1.468	.202	-.467	1.711
a. DV: BI								

Moderation Analysis for BI 

A moderation analysis was conducted to examine whether certain variables, such as age, comfort with technology, SE, and anxiety, moderate the effects of IVs such as PE, EE, SI, and FC on the DV and BI. This involved computing Z-scores for all variables using descriptive statistics and creating multiplicative interaction terms for analysis. 

The moderation analysis revealed no significant effects on the relationships between PE and BI or SI and BI based on age, experience, comfort with technology, gender, anxiety, or SE. Although FC significantly enhanced BI (β=0.63, p≤0.05), the positive impact was diminished by the moderators such as comfort with technology (β=-3.8, p=0.01), gender (β=-0.43, p=0.02), and SE (β=-3.5, p=0.02). No moderation was observed for anxiety, experience, and age.

*Moderation Analysis For AT*****

The moderation analysis revealed that age significantly moderates the relationship between PE and AT, weakening this linkage as age increases (β=-0.67, p=0.04). No moderating effects of experience, comfort with technology, gender, anxiety, or SE were observed between PE and AT. For EE and AT, results showed a positive impact of EE on AT (β=0.66, p=0.05), with gender significantly enhancing this effect (β=0.81, p=0.04), indicating a moderating role of gender. FC did not impact AT or any tested moderators (age, experience, comfort with technology, gender, anxiety, and SE). SI initially had a positive relationship with AT (β=0.68, p=0.02). However, moderation analysis indicated significant negative moderating effects of age (β=-0.67, p=0.03), comfort with technology (β=-0.84, p=0.03), and experience (β=-0.91, p=0.01), reversing the positive impact of SI on AT.

Qualitative findings

**Figure 7:**
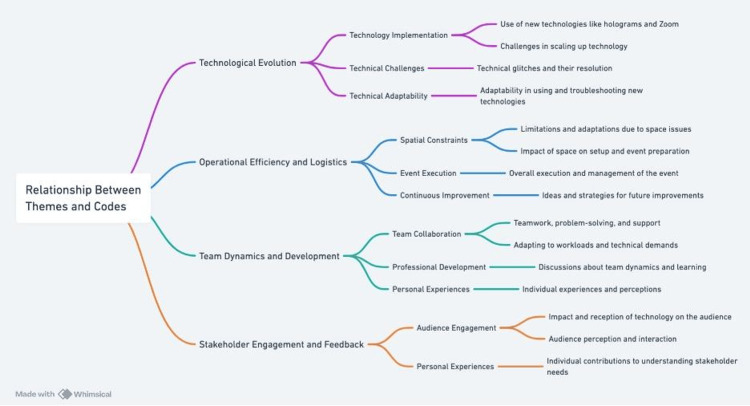
Interrelationships of the codes and themes

Following Braun et al., we constructed four themes from the focus group discussion, namely, 1) technical evolution and adaptation, 2) operational efficiency and logistics, 3) team dynamics and development, and 4) stakeholders' engagement and feedback (Figure 7) [19].

Technological Evolution 

This theme comprised the complexities and solutions in integrating advanced HDT with existing systems and processes. Individual codes included technological implementation, technical challenges, and technical adaptability. Participants discussed encountering technological challenges during the setup and execution of HDT activity and the circumnavigation around those challenges. One participant shared, “For challenges with using this technology, I would say installations and implementation of the technology itself (IT1),” and “We identified some of the challenges with the solution from [the] Proto [unit], which pushed us then to be using Zoom (IT3).” Another participant shared, “It is not really designed for two individuals (IT9),” and “There are some limitations trying to experience that technology via Zoom doesn't show its full capabilities in my opinion (IT2).” Another shared, “I think we put a good amount of time in testing and finding a scenario that would work (IT5).”

Operational Efficiency and Logistics 

This theme revolved around the logistical and practical aspects of implementing the HDT in educational settings. It included space requirements, setup complexities, and the impact on daily routines and work processes. Because participants belonged to the IT department, they focused on efficient logistics. They shared, “We had a space that maybe was not ideal for everything ... but I think we worked through the challenges and kinda just kept things going (IT7).” Another participant shared, “With a portable type setup, 50% of the time for the event could be equal to the time for setup and possibly even tear down (IT8). The proto unit is pretty heavy and requires a team to at least move it around (IT5).” The team shared that because of their IT background, the activity was implemented efficiently, “Getting everything set up for the beaming kit went straightforward, and we were to get that done in good time (IT4).” For future applications, they shared, “I think that is very innovative, and I could see the use for it in other areas (IT2).” Regarding preparation, they shared that they had allotted time in their schedule and had been preparing for months for HDT simulation implementation, which made it easier when the time came. 

Team Dynamics and Development

The IT expressed the cohesiveness of their team in the face of challenges. “It's an increased workload. But also, to an extent, we've grown our team as well (IT2).” They worked together to overcome the challenges, as shown by these quotes: “We had really less than 24 hours in the space to get everything up and running and tested (IT10),” “We made a quick change and something worked (IT9),” and “We did a lot of things quickly in the other campus yesterday, but also this morning we had about 14 members doing the rest of the cleanup today.” (IT5)

Stakeholders' Engagement and Feedback

This theme addressed how the target audience, the students and faculty, interacted with and perceived the new technology observed by IT. It included the target audience’s initial reactions, ranging from excitement and appreciation for the innovation to skepticism and preference for traditional methods. The IT team members were either present in person or observed the activity live using videoconferencing. Therefore, they could observe the audience and their interaction with the HDT and among themselves during the activity. They observed, “When the kit was in and in the live beaming mode, I think the students got a big reaction from that versus the pre-recorded videos (IT4)”. Also, “We got almost 50% at least, what I heard from some of the students, like Oh that's really cool. And then others were like I wouldn't know better than looking at the screen (IT7)”. They noticed, “I think that innovation is there, the thing that the students see something different that they are motivated to see okay what is going on (IT3).”

## Discussion

Our study evaluated the acceptability and practicality of HDT among IT personnel through the UTAUT model and focus group discussion. It uncovered alignment and discrepancies across UTAUT constructs, as illustrated through qualitative and quantitative analyses. Linear regression identified that PE and FC did not directly affect BI or AT but indirectly affected these outcomes through EE and SI. Qualitative analysis corroborated these findings, suggesting BI and AT depend on multiple factors. The study highlights the role of UTAUT constructs in facilitating HDT adoption, with significant implications for technology acceptance, emphasizing the importance of technological evolution, team dynamics, and stakeholder engagement (Table 4).

**Table 4:** Alignments of qualitative themes with key quantitative factors The check mark ✓ sign shows the overlap between quantitative and qualitative findings, while a negative (-) sign points toward the lack of overlap between those findings. PE, performance expectancy; EE, effort expectancy; SI, social influence; FC, facilitating conditions

Variables/themes	Technological evolution	Operational efficiency and logistics	Team dynamics and development	Stakeholders' engagement and feedback
PE	✓	(-)	(-)	✓
EE	✓	(-)	(-)	(-)
SI	(-)	(-)	✓	✓
FC	(-)	✓	(-)	(-)

The theme of technological evolution highlighted IT professionals' resilience and adaptability, demonstrating that despite initial challenges, effective internal support and IT practices enhance new technology adoption, a phenomenon that parallels the quantitative insights of PE and EE. It is logical to infer that technological evolution correlates with performance and EE. This alignment suggests that technological advances and adaptations are likely influenced by how much effort and performance individuals expect from this technology, which, in turn, is supported by the literature that infrastructure availability, institutional support, and organizational resources improve technology integration [23]. Participants benefited from leadership support, allocating specific project time within their roles as IT providers, preparing them to tackle technological issues effectively, and thus prompting the natural evolution of technology adoption. Moreover, understanding HDT adoption can extend its applications in healthcare, including diagnostics and telemedicine [4].

The operational efficiency and logistics theme emphasizes the importance of logistical planning and IT skills for the practical challenges of HDT implementation, including space and setup complexities. This aligns with quantitative analysis showing that technological, logistical, and infrastructure support positively impacts HDT adoption and use. This is consistent with literature indicating that holographic simulations demand planning and resources beyond traditional simulations [2,4]. Literary evidence advocates for comprehensive planning and rehearsals to address logistical issues [14]. Effective support in these simulations is achieved through multiple rehearsals and feedback integration, leading to a well-coordinated final rehearsal with all involved parties.

The team dynamics and development theme complements "operational efficiency and logistics," as evolving team dynamics help tackle logistical challenges. The link between these themes is supported by quantitative findings on the IT team's EE and SI enhancing the intention to use HDT, highlighting how SI and FC foster a conducive environment for technology adoption and integration, thereby boosting team performance and acceptance. Unexpectedly, team development in overcoming technical obstacles happened, signifying the cohesive dynamics for operational success. Akinnuwesi et al. highlight that SIs, both internal and external, affect team performance, with leadership support being crucial for positive dynamics and efficiency [23]. In the current HDT use case, administrative support enhanced mutual assistance and collaboration, contributing to positive SI on the team and effective team dynamics, which are crucial for overcoming operational challenges.

*Stakeholder Engagement and Feedback* revealed a divergence in perceptions. Qualitative data indicated a range of reactions from enthusiasm to skepticism, while quantitative data showed no significant association between AT and BI with PE and SI. The moderating factors influencing the relationship between IV included age, experience, comfort with technology, gender, anxiety, and SE. This disparity may stem from the participants' roles as IT department employees accustomed to various technologies and thus less influenced by the social aspects of HDT. A key finding was the negative correlation between SE and PE. Individuals with higher SE tended to perceive lower PE from the HDT. This suggests that those confident in their abilities might set higher standards or expectations for the technology's performance, leading to a perception that the technology falls short of their expectations. Similarly, a negative correlation was observed between comfort with technology and FC, indicating that individuals more comfortable with technology might not perceive FC as necessary or enhancing their performance with HDT, relying instead on their own capabilities.

Furthermore, there was a negative correlation between comfort with technology and anxiety, demonstrating that individuals more at ease with technology experience less anxiety and stress when using HDT. This finding aligns with the intuitive understanding that familiarity and confidence with technology typically reduce anxiety [24]. The negative correlation between AT and experience suggests that more experienced individuals might have a less favorable attitude toward new technology like HDT. This could be attributed to their established routines and familiarity with existing technologies, making them less open to adopting new tools [24].

These diverging factors highlight the complex dynamics between individual characteristics, user perceptions, and attitudes toward new technology. The study highlights the importance of considering these moderating factors when introducing and implementing new technologies in educational settings. Understanding these relationships, especially from an administrative perspective for educational settings, can inform better strategies for technology adoption, ensuring that support structures are tailored to meet users' diverse needs. Additionally, simulation learners exhibited skepticism and intrigue, indicating a need for interaction through HDT, as Diaz et al. suggested [2]. Developing customized communication and engagement strategies is crucial to narrowing the gap between the potential of technology and stakeholder acceptance. Although not explicitly indicated in quantitative findings, such strategies are vital for successfully integrating HDT in healthcare settings. Future research should continue to explore these dynamics in various contexts to develop a comprehensive understanding of technology adoption and integration in healthcare education.

Future research

This discussion highlights the need for further experimental, multisite, and longitudinal research to understand these causal links and strengthen the UTAUT model's capacity to accurately predict technology adoption and use. More research, especially in different locations and with various learning content, especially in the patient care setting, is necessary to understand the full benefits and applicability of HDT in healthcare education and practice.

Strengths and limitations

The study effectively combines qualitative and quantitative approaches, enriching the data and describing HDT acceptance among IT staff. Validity from realistic simulations across campuses, in-person and online, captures complex user experiences similar to real life. Through meticulous scenario development and thorough pre-simulation tests, the collaborative efforts between the simulation faculty and the IT department further strengthen the study. Additionally, IT personnel with prior simulation experience beyond HDT provided key insights into the benefits, logistics, and practical aspects of HDT simulation. 

However, the study's limitations include a small sample size of a homogenous IT population from a single university, which reduces generalizability. Factors such as the specific holographic models used, their integration with IT systems, and the dynamics of faculty-IT relationships could influence the results. Furthermore, the technical complexity of setting up the holographic technology raises questions about its feasibility and adaptability to other environments.

## Conclusions

Our study on HDT among IT personnel demonstrates its feasibility for health professions education, highlighting the importance of PE, EE, SI, and FC. The HDT shows promise in enhancing learning outcomes with immersive 3D visuals and interactivity, provided that certain conditions are met. The feasibility of HDT adoption for IT teams depends on simplifying the technology's use, ensuring robust stakeholder engagement, including institutional support, fostering positive SI, and addressing diverse user experience levels. Future research should explore its broader applicability in health and education sectors, emphasizing technology acceptance and strategic implementation. Overall, this research emphasizes the multifaceted nature of technology acceptance, guiding strategies for more effective implementation and integration of innovative technologies like HDT. 

## Appendices

**Table 5:** UTAUT survey question UTAUT, Unified Theory of Acceptance and Use of Technology

UTAUT post-intervention survey questions	
Directions: provide your opinion on a scale of 1 through five, where 1 - strongly disagree; 2 - disagree; 3 - neutral; 4 - agree; 5 - strongly agree	
Performance expectancy	
U2:	Using holographic technology would improve my job performance
U6:	I would find holographic technology useful in my job
RA1:	Using holographic technology would enable me to accomplish tasks more quickly
RA2:	Using holographic technology would improve the quality of my work
RA5:	Using holographic technology would increase my productivity
OE2:	If I use holographic technology, I will spend less time on routine job tasks
OE7:	If I use holographic technology, I will increase my chances of getting a raise
Effort expectancy	
EOU3:	My interaction with the holographic technology would be clear and understandable.
EOU5:	It would be easy for me to become skillful at using holographic technology
EOU6:	I would find holographic technology easy to use
EU4:	Learning to operate holographic technology would be easy for me
C1:	Using holographic technology would take too much time from my normal duties
Social influence	
SN1:	People who influence my behavior think that I should use this new technology
SN2:	People who are important to me think that I should use this new technology
SF2:	The senior management of this university has been helpful in using this new technology
SF4:	In general, the organization has supported the use of this new technology
I3:	Having this new technology is a status symbol in my organization
Facilitations conditions	
PBC2:	I have the resources necessary to use this new technology
PBC3:	I have the knowledge necessary to use this new technology
PBC5:	This new technology is not compatible with other systems (techniques and technologies) I use
FC3:	A specific person (or group) is available to assist with difficulties while using this new technology
C2:	I think that using this new technology fits well with the way I like to work
Attitude toward using technology	
Al:	Using holographic technology is a good idea
AF1:	holographic technology makes work more interesting
AF2:	Working with holographic technology would be fun
Affect1:	I like working with holographic technology
Self-efficacy	
I could complete a job or task using holographic technology ...	
SE1:	If there was no one around to tell me what to do as I go
SE4:	If I could call someone for help if I got stuck
SE6:	If I had a lot of time to complete the job for which the software was provided
SE7:	If I had just the built-in help facility for assistance
Anxiety	
ANX1:	I feel apprehensive about using this new technology
ANX2:	It scares me to think that I could lose a lot of information by hitting the wrong key
ANX3:	I hesitate to use this new technology for fear of making mistakes I cannot correct
ANX4:	This new technology is somewhat intimidating to me
Behavioral intention	
BI1:	I intend to use h-tech in the next months
B12:	I predict I would use h-tech in the next months
B13:	I plan to use h-tech in the next months

**Table 6:** UTAUT definitions of the constructs UTAUT, Unified Theory of Acceptance and Use of Technology

UTAUT definitions of the constructs		
Main constructs/determinants/moderators	Root construct components	Definitions
Root constructs/determinants		
1. Performance expectancy (the strongest predictor of intention to use)	U: Perceived usefulness; U1-U6	The degree to which a person believes that using a particular system would enhance his or her job performance
	EM: Extrinsic motivation; EM1-EM6	The perception that users will want to perform an activity because it is perceived to be instrumental in achieving valued outcomes that are distinct from the activity itself such as improved job performance, pay, or promotions
	JF: Job fit; JF1-JF6	How the capabilities of a system enhance an individual’s job performance
	RA: Relative advantage; RA1-RA5	The degree to which using innovation is perceived as being better than using its precursor
	OE: Outcome expectation; OE1-OE7	Relates to the consequences of the behavior
2. Effort expectancy	pEOU: Perceived ease of use; EOU1-EOU7	The degree to which a person believes that using a system would be free of effort
	C: Complexity	The degree to which a system is perceived as relatively difficult to understand and use
	EOU: Ease of use; EOU1- EOU4	The degree to which using innovation is perceived as being difficult to use
3. Social influence	SN: Subjective norm; SN1-SN2	The person’s perception that most people who are important to him think he should or should not perform the behavior in question
	SF: Social factors; SF1-SF4	The individual’s internalization of the reference group’s subjective culture, specific interpersonal agreements that the individual has made with others in specific social situations
	I: Image I1-I3	The degree to which the use of an innovation is perceived to enhance one’s image or status in one’s special system
4. Facilitating conditions	BC: Perceived behavioral control; BC1-BC5	Reflects perceptions of internal and external constraints on behavior and encompasses self-efficacy, resource-facilitating conditions, and technology-facilitating conditions
	FC: Facilitating conditions; FC1-FC3	Objective factors in the environment that observers agree make an act easy to do, including the provision of computer support
	C: Compatibility; C1-C3	The degree to which an innovation is perceived as being consistent with existing values, needs, and experiences of potential adopters
Moderators impacting intentions to use directly or indirectly		
Age		We collected age information as part of the demographics
Gender		We collected gender information as part of the demographics
Experiences		We collected previous technological experience information as part of the demographics
Voluntariness		The degree to which the use of the technology is perceived as being optional or voluntary by the user versus being mandatory
Attitude towards technology	A: Attitude towards behavior; A1-A4	An individual’s positive or negative feelings about performing the target behavior
	Intrinsic motivation; IM1-IM3	The perception that users will want to perform an activity for no apparent reinforcement other than the process of performing the activity per se
	Affect towards use; AU1-AU3	Feelings of joy, elation, or pleasure; or depression, disgust, displeasure, or hate associated by an individual with a particular act
	Affect affect1; Affect5	An individual’s liking of the behavior
Self-efficacy	SE	The belief in one's capabilities to organize and execute the courses of action required to manage prospective situations
Anxiety	ANX	Anxiety represents the level of unease or fear associated with using technology
Behavioral Intention	BI	The degree of an individual's intention to use the technology
